# Outcomes After Metal-on-metal Hip Revision Surgery Depend on the Reason for Failure: A Propensity Score-matched Study

**DOI:** 10.1007/s11999.0000000000000029

**Published:** 2018-01-17

**Authors:** Gulraj S. Matharu, Andrew Judge, David W. Murray, Hemant G. Pandit

**Affiliations:** G. S. Matharu, A. Judge, D. W. Murray, H. G. Pandit, Nuffield Department of Orthopaedics, Rheumatology and Musculoskeletal Sciences, University of Oxford, Nuffield Orthopaedic Centre, Oxford, UK; A. Judge, MRC Lifecourse Epidemiology Unit, Southampton General Hospital, University of Southampton, Southampton, UK; H. G. Pandit, Leeds Institute of Rheumatic and Musculoskeletal Medicine, Chapel Allerton Hospital, Leeds, UK

## Abstract

**Background:**

Metal-on-metal hip replacement (MoMHR) revision surgery for adverse reactions to metal debris (ARMD) has been associated with an increased risk of early complications and reoperation and inferior patient-reported outcome scores compared with non-ARMD revisions. As a result, early revision specifically for ARMD with adoption of a lower surgical threshold has been widely recommended with the goal of improving the subsequent prognosis after ARMD revisions. However, no large cohorts have compared the risk of complications and reoperation after MoMHR revision surgery for ARMD (an unanticipated revision indication) with those after non-ARMD revisions (which represent conventional modes of arthroplasty revision).

**Questions/purposes:**

(1) Does the risk of intraoperative complications differ between MoMHRs revised for ARMD compared with non-ARMD indications? (2) Do mortality rates differ after MoMHRs revised for ARMD compared with non-ARMD indications? (3) Do rerevision rates differ after MoMHRs revised for ARMD compared with non-ARMD indications? (4) How do implant survival rates differ after MoMHR revision when performed for specific non-ARMD indications compared with ARMD?

**Methods:**

This retrospective observational study involved all patients undergoing MoMHR from the National Joint Registry (NJR) for England and Wales subsequently revised for any indication between 2008 and 2014. The NJR achieves high levels of patient consent (93%) and linked procedures (ability to link serial procedures performed on the same patient and hip; 95%). Furthermore, recent validation studies have demonstrated that when revision procedures have been captured within the NJR, the data completion and accuracy were excellent. Revisions for ARMD and non-ARMD indications were matched one to one for multiple potential confounding factors using propensity scores. The propensity score summarizes the many patient and surgical factors that were used in the matching process (including sex, age, type of primary arthroplasty, time to revision surgery, and details about the revision procedure performed such as the approach, specific components revised, femoral head size, bearing surface, and use of bone graft) using one single score for each revised hip. The patient and surgical factors within the ARMD and non-ARMD groups subsequently became much more balanced once the groups had been matched based on the propensity scores. The matched cohort included 2576 MoMHR revisions with each study group including 1288 revisions (mean followup of 3 years for both groups; range, 1-7 years). Intraoperative complications, mortality, and rerevision surgery were compared between matched groups using univariable regression analyses. Implant survival rates in the non-ARMD group were calculated for each specific revision indication with each individual non-ARMD indication subsequently compared with the implant survival rate in the ARMD group using Cox regression analyses.

**Results:**

There was no difference between the ARMD and non-ARMD MoMHR revisions in terms of intraoperative complications (odds ratio, 0.97; 95% confidence interval [CI], 0.59-1.59; p = 0.900). Mortality rates were lower after ARMD revision compared with non-ARMD revision (hazard ratio [HR], 0.43; CI, 0.21-0.87; p = 0.019); however, there was no difference when revisions performed for infection were excluded from the non-ARMD indication group (HR, 0.69; CI, 0.35-1.37; p = 0.287). Rerevision rates were lower after ARMD revision compared with non-ARMD revision (HR, 0.52; CI, 0.36-0.75; p < 0.001); this difference persisted even after removing revisions performed for infection (HR, 0.59; CI, 0.40-0.89; p = 0.011). Revisions for infection (5-year survivorship = 81%; CI, 55%-93%; p = 0.003) and dislocation/subluxation (5-year survivorship = 82%; CI, 69%-90%; p < 0.001) had the lowest implant survival rates when compared with revisions for ARMD (5-year survivorship = 94%; CI, 92%-96%).

**Conclusions:**

Contrary to previous observations, MoMHRs revised for ARMD have approximately half the risk of rerevision compared with non-ARMD revisions. We suspect worldwide regulatory authorities have positively influenced rerevision rates after ARMD revision by recommending that surgeons exercise a lower revision threshold and that such revisions are now being performed at an earlier stage. The high risk of rerevision after MoMHR revision for infection and dislocation is concerning. Infected MoMHR revisions were responsible for the increased mortality risk observed after non-ARMD revision, which parallels findings in non-MoMHR revisions for infection.

**Level of Evidence:**

Level III, therapeutic study.

## Introduction

Approximately 1.5 million large-diameter metal-on-metal hip replacements (MoMHRs) have been implanted worldwide in the form of stemmed THAs and hip resurfacings. However, MoMHRs have experienced unexpected high short-term revision rates [26, 27]. Revision surgery for MoMHRs can be indicated for problems like dislocation, loosening, infection or fracture, or for an unanticipated complication termed adverse reactions to metal debris (ARMD) [10, 13, 20, 21]. ARMD can cause large, destructive periprosthetic masses, which often are treated with revision [10, 13, 21]. Although worldwide regulatory authorities have subsequently advised against the future use of most MoMHR devices, patients with these implants in situ require followup to help in the early detection of complications [15, 18, 28].

Despite the high revision rates of MoMHRs [26, 27], at least 80% of these implants remain in situ [5, 20]. However, little is known about the risk of complications and reoperation after MoMHR revision surgery, especially when performed for ARMD [17]. Early observations suggested that half of the patients revised for ARMD sustained major complications, and more than one-third underwent reoperation [10]. Similar observations were reported in subsequent small cohorts [19, 23]. Furthermore, ARMD revision surgery has been associated with an increased risk of early complications and reoperation and inferior patient-reported outcome scores compared with MoMHR revisions performed for non-ARMD indications [10]. The poor prognosis after ARMD revision was thought to be the result of the invasive and destructive nature of these lesions [10, 13, 21]. This led surgeons and regulatory authorities to widely recommend performing early revision in MoMHRs with ARMD [7, 10, 18, 28]. Surgeons subsequently adopted a lower threshold for performing revision specifically for ARMD with the goal of improving the subsequent prognosis after these ARMD revision procedures [7, 10, 14]. Given many young and active patients undergoing MoMHR are likely to require revision surgery for ARMD in the future [5, 14, 20], it is important to have robust information about the risk of complications and reoperation after this unanticipated revision indication compared with patients undergoing MoMHR revisions for non-ARMD indications, which represent the conventional modes of arthroplasty revision. However, no large cohorts have compared the risk of complications and reoperation after MoMHR revision surgery for ARMD with those after non-ARMD revisions.

The National Joint Registry (NJR) for England and Wales was established in April 2003 to identify poorly performing implants early [20]. It is the world’s largest arthroplasty registry, containing details of two million joint replacement procedures. We assessed a large patient cohort from the NJR who all underwent MoMHR revision surgery and asked the following questions: (1) Does the risk of intraoperative complications differ between MoMHRs revised for ARMD compared with non-ARMD indications? (2) Do mortality rates differ after MoMHRs revised for ARMD compared with non-ARMD indications? (3) Do rerevision rates differ after MoMHRs revised for ARMD compared with non-ARMD indications? (4) How do implant survival rates differ after MoMHR revision when performed for specific non-ARMD indications compared with ARMD?

## Materials and Methods

A retrospective observational cohort study was performed using NJR data. The NJR records all hip replacements performed at all hospitals in England and Wales. Patients provide voluntary consent for their personal details to be recorded within the NJR, which is typically obtained before undergoing surgery. These unique patient identifiers subsequently allow linkage of primary hip replacements to any future surgical procedures in which components are removed or exchanged. The NJR achieves high levels of patient consent (93%) and procedure linkage (95%) [20]. Furthermore, recent validation studies have demonstrated that when revision procedures have been captured within the NJR, the data completion and accuracy were excellent [24, 25]. Before obtaining the data set, the NJR database was linked with the Office for National Statistics (ONS) database using unique patient identifiers. The ONS provides data on all-cause mortality.

The study protocol was approved after submission of a formal application to the NJR Research Sub-Committee. Because patients provide informed consent for inclusion of their data in the NJR, further approvals such as from the institutional review board were not required for this study. Anonymized patient data were extracted from the NJR on August 14, 2015. This data set contained details of all primary MoMHRs (MoM THAs and hip resurfacings) recorded in the NJR in patients who subsequently underwent revision surgery for any indication between August 1, 2008, and August 14, 2014 (n = 5867). The former date represents when the NJR introduced the term adverse soft tissue reaction to particulate debris on the data capture forms as a revision indication for surgeons to select. We have elected to classify this revision indication as ARMD throughout, given that this is currently the most commonly used and accepted term in the literature [13]. Before this term was added to the NJR data capture forms, ARMD revisions were either undiagnosed or incorrectly recorded using non-ARMD indications. The latter study date allowed a minimum 1-year followup for assessing the study endpoints after revision.

**Fig. 1:**
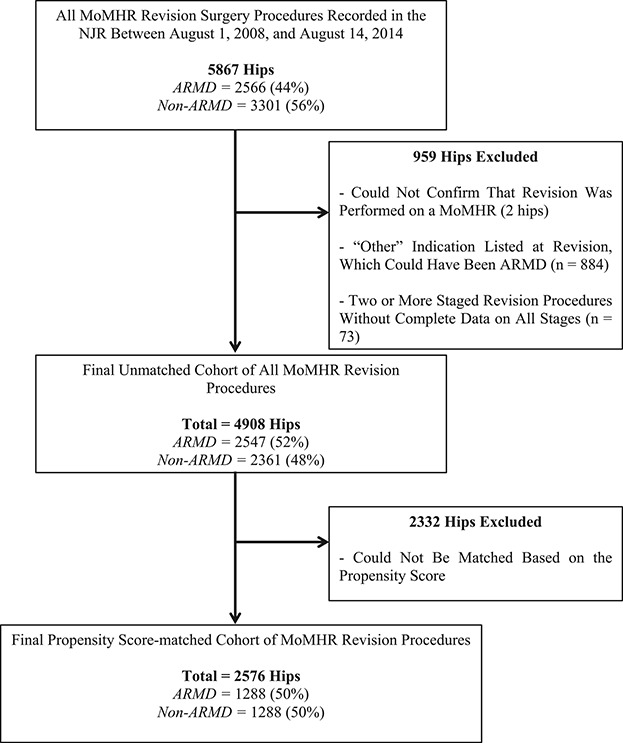
A flowchart illustrating the selection criteria used in this study.

Hip revisions were excluded if it was not possible to confirm that the primary hip replacement had a MoM bearing surface (n = 2) and when incomplete data were available for revision procedures involving two or more stages (n = 73) (Fig. 1). We also excluded all MoMHR revisions in which the intraoperative findings included “other” but ARMD was not recorded (n = 884). Because recognition of ARMD as a clinical problem associated with MoMHRs occurred over time [10, 13, 21], it was possible that some or all of these “other” patients may have had undiagnosed ARMD or incorrectly recorded ARMD. This left 4908 patients with primary MoMHRs (2913 MoM THAs and 1995 hip resurfacings) undergoing revision surgery for any indication (Fig. 1).

This study was specifically designed to assess the effect of MoMHR revision indication on the subsequent risk of intraoperative complications, mortality, and rerevision. Despite using a large registry cohort, the number of events for each endpoint was suspected to be relatively small. Adjusting for all patient and surgical covariates was likely to lead to overfitting of any statistical models with other covariates having the potential to incorrectly influence the findings between revision indication and the subsequent risk of intraoperative complications, mortality, and rerevision. Furthermore, baseline characteristics differed substantially between the ARMD and non-ARMD groups (Table 1).

**Table 1.**
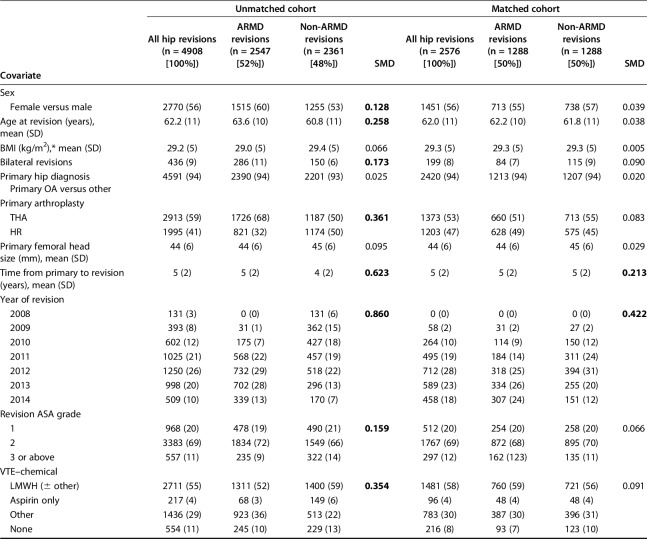

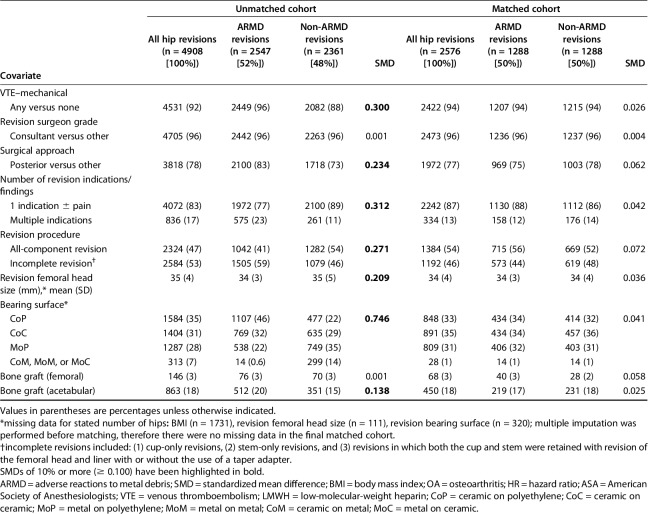
Patient and surgical factors before and after propensity score matching

Given the potential for confounding by revision indication to affect comparison of the study endpoints, revision procedures for ARMD and non-ARMD indications were matched for multiple potential confounding factors using propensity score techniques [3, 9]. The propensity score summarizes the numerous patient and surgical factors that were used in the matching process (detailed subsequently) using one single score for each revised hip. The patient and surgical factors within the ARMD and non-ARMD groups subsequently became much more balanced once the groups had been matched based on the propensity scores, which in turn should improve the validity of the study findings. Matching was performed using a one-to-one ratio. The algorithm used matched on the logit of the propensity score with a 0.02-SD caliper width. We used greedy matching (that is, each ARMD revision patient was matched to the nearest non-ARMD revision patient) without replacement (once a match is made, that specific patient was no longer available as a potential match for subsequent patients), which has been shown to have superior performance for estimating treatment effects [3]. ARMD and non-ARMD groups were matched for all covariates listed (Table 1), including age, sex, body mass index (BMI), primary arthroplasty, year of revision, American Society of Anesthesiologists grade, surgeon grade, surgical approach, the number of revision indications recorded by the operating surgeon, and components implanted including bearing surface and femoral head size. The final matched cohort for analysis included 2576 MoMHR revision procedures with 1288 in the ARMD group (660 THAs and 628 hip resurfacings) and 1288 in the non-ARMD group (713 THAs and 575 hip resurfacings) (Fig. 1). The mean followup from revision surgery for both groups was 3 years (range, 1-7 years).

All surgeons in England and Wales complete a data capture form after performing any primary or revision hip arthroplasty. These forms are subsequently submitted to the NJR and entered into their database. For all arthroplasty procedures, the NJR collects data on patient demographics and the surgical procedure, which were all used in the matching process (see previously and Table 1). In addition, NJR data for all revision procedures included details of the one or more intraoperative findings recorded by the operating surgeon (ARMD, infection, fracture, loosening, lysis, dislocation, subluxation, implant malalignment, implant mismatch, cup wear, implant fracture, liner dissociation, pain, and other).

The binary study exposure was whether a MoMHR revision was performed for ARMD or non-ARMD indications with hips in each group matched as described. Further analysis was performed with the non-ARMD group subdivided into specific revision indications. When multiple revision indications were recorded, the following hierarchy was used, which was developed using previous studies [11, 12]: (1) ARMD; (2) infection; (3) fracture; (4) loosening and/or lysis; (5) dislocation/subluxation; (6) other (includes implant malalignment/mismatch, cup wear, implant fracture, and liner dissociation); and (7) unexplained pain. Revisions for fracture and loosening and/or lysis included revisions of the acetabular and/or femoral side [11, 12].

The three study endpoints of interest were intraoperative complications during MoMHR revision (including calcar crack, pelvic and/or femoral shaft penetration, trochanteric and/or femoral shaft fracture, and other complications), all-cause mortality, and all-cause rerevision surgery after revision. Implant survival rates in the non-ARMD group were also determined for each specific revision indication with each individual non-ARMD indication subsequently compared with the implant survival rate after ARMD revision.

### Statistical Analysis

There was missing data for three covariates (BMI, revision femoral head size, revision bearing surface). BMI contained a significant proportion of missing values as well as a number of implausible values (such as 0 or 1 kg/m^2^). The NJR does collect information on the arthroplasty components implanted. However, data on femoral head size and bearing surface at the MoMHR revision procedure were not consistently available (either truly missing or ambiguous and therefore considered missing). This most notably occurred when incomplete revision procedures were performed (ie, when one or more components from the primary MoMHR was retained at revision). Multiple imputation methods were used to provide estimates for the missing data values in the three covariates with incomplete data with a total of 50 imputed data sets generated. Imputation models included all other covariates with complete data available as well as the study endpoints (including Nelson-Aalen estimate for survival models) given they all carried information about the missing covariate values. As a sensitivity analysis, the study endpoints were also assessed using a complete case data set, which excluded BMI only given the large proportion of missing BMI data (35%) (see Appendix, Supplemental Digital Content 1).

Logistic regression was used to generate a propensity score, representing the probability that a MoMHR was revised for ARMD. ARMD and non-ARMD revisions were subsequently matched based on the propensity score. Standardized mean differences (SMDs) were examined both before and after matching to assess for any covariate imbalance between ARMD and non-ARMD revisions with SMDs of ≥ 10% considered suggestive of covariate imbalance [2].

Cumulative patient and implant survival rates after MoMHR revision were determined using the Kaplan-Meier method. The endpoint for implant survival was rerevision surgery (any component removal or exchange). Patients not undergoing rerevision or death were censored on the study end date (August 14, 2015). The study endpoints after revision surgery were compared between ARMD and non-ARMD groups using logistic (intraoperative complications) and Cox (mortality and rerevision) regression models. To account for clustering within the matched cohort, a robust variance estimator was used in the Cox regression models with a conditional logistic regression model used for assessing intraoperative complications [4]. For Cox regression, proportional hazards assumptions were assessed using Schoenfeld’s residuals. Univariable regression models were assessed for the matched cohort as well as adjusted models. These adjusted models accounted for any residual covariate imbalance after matching, which was defined as an SMD of ≥ 10% for any covariate after matching. One of the non-ARMD indications for revision was infection, which can be associated with higher mortality [6, 30]. As a sensitivity analysis, these regression analyses were therefore repeated in a matched cohort of patients in which MoMHR revisions performed for infection had been excluded.

We used R (Version 3.1.2; R Foundation for Statistical Computing, Vienna, Austria) to perform the propensity score matching and Stata (Version 13.1; College Station, TX, USA) for all other analyses. Probability values < 0.05 and 95% confidence intervals (CIs) were used.

## Results

There was no difference between the ARMD (2.4%) and non-ARMD (2.5%) MoMHR revisions in terms of the risk of intraoperative complications (odds ratio, 0.97; CI, 0.59-1.59; p = 0.900; Table 2). The most common intraoperative complications in both groups were calcar and greater trochanteric fractures. A regression model adjusting for two covariates with residual imbalance after matching (time from primary to revision and year of revision) produced similar results as the unadjusted models (Table 2). When revisions performed for infection were excluded from the non-ARMD group, there was still no difference in the risk of intraoperative complications between matched ARMD and non-ARMD revisions (odds ratio, 1.00; CI, 0.58-1.82; p = 1.00; see Appendix, Supplemental Digital Content 2).

**Table 2.**
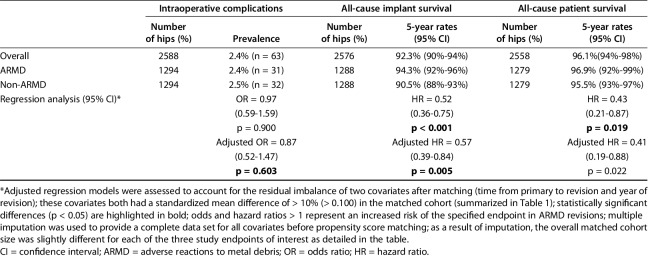
Intraoperative complications, implant survival, and patient survival after metal-on-metal hip replacement revision surgery in the matched cohort

All-cause mortality rates were lower after ARMD revision compared with non-ARMD revision (Table 2). The 5-year cumulative all-cause patient survival rate after ARMD revision was 96.9% (CI, 92%-99%) compared with 95.5% (CI, 93%-97%) after non-ARMD revision (hazard ratio [HR], 0.43; CI, 0.21-0.87; p = 0.019). However, when revisions performed for infection were excluded from the non-ARMD group, there was no difference in mortality rates after ARMD revision compared with matched non-ARMD revisions (HR, 0.69; CI, 0.35-1.37; p = 0.287; see Appendix, Supplemental Digital Content 2). The overall all-cause mortality risk was 1.6% (n = 41) with a mean time from revision to death of 2 years (range, 0.1-6 years). A regression model adjusting for two covariates with residual imbalance after matching (time from primary to revision and year of revision) produced similar results as the unadjusted models (Table 2).

**Fig. 2A-B:**
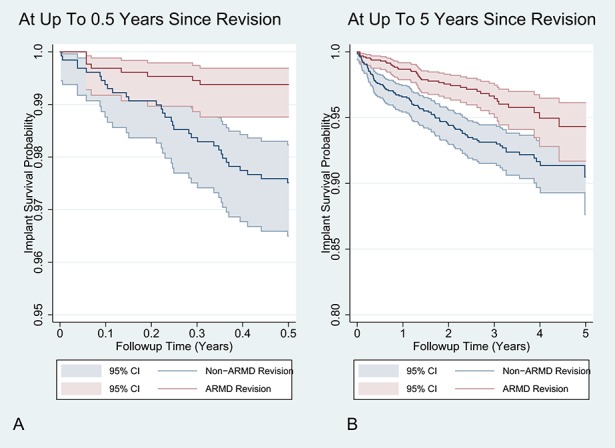
A Kaplan-Meier graph illustrating the cumulative all-cause implant survival rate after revision surgery performed for ARMD indications compared with non-ARMD indications. The shaded area represents the respective upper and lower limits of the 95% CIs. During the first 0.5 years after revision surgery, the proportional hazards assumption was not satisfied (**A**) with the non-ARMD revision group having a disproportionally higher rate of rerevision compared with the ARMD group. From 0.5- to 5-year followup from revision surgery, the proportional hazards assumption was satisfied (**B**).

All-cause rerevision rates were lower after ARMD revision compared with non-ARMD revisions (Table 2). The 5-year cumulative all-cause implant survival rate after ARMD revision was 94.3% (CI, 92%-96%) compared with 90.5% (CI, 88%-93%) after non-ARMD revision (HR, 0.52; CI, 0.36-0.75; p < 0.001; Fig. 2). The overall all-cause rerevision risk was 5% (n = 134). Mean time from revision to rerevision was 1.4 years (range, 0.003-6 years). Rerevision indications included dislocation/subluxation (21%; n = 28), ARMD (19%; n = 26), infection (17%; n = 23), loosening and/or lysis (17%; n = 23), other (11%; n = 15), unexplained pain (8%; n = 10), and fracture (7%; n = 9). A regression model adjusting for two covariates with residual imbalance after matching (time from primary to revision and year of revision) produced similar results to the unadjusted models (Table 2). When revisions performed for infection were excluded from the non-ARMD group, ARMD revisions continued to have lower rerevision rates compared with matched non-ARMD revisions (HR, 0.59; CI, 0.40-0.89; p = 0.011; see Appendix, Supplemental Digital Content 2).

Compared with ARMD revision, implant survival rates were lower when MoMHR revisions were performed for infection, fracture, loosening and/or lysis, dislocation/subluxation, and unexplained pain (Table 3). Revisions performed for infection (81.2%; CI, 55%-93%; p = 0.003) and dislocation/subluxation (81.9%; CI, 69%-90%; p < 0.001) had the lowest 5-year implant survival rates when compared with revisions for ARMD (94.3%; CI, 92%-96%).

**Table 3.**
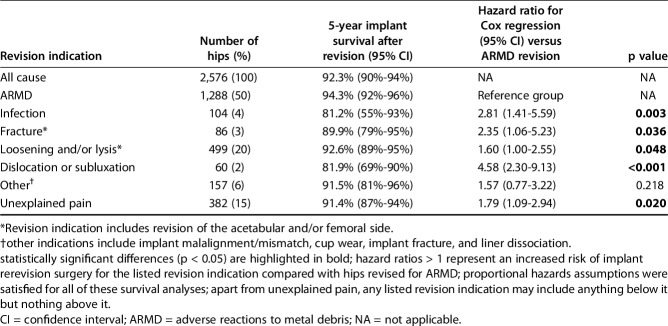
Comparison of implant survival rates by specific metal-on-metal hip revision indication

## Discussion

Early studies observed MoMHR revisions performed for ARMD had a high risk of rerevision, poor patient-reported outcomes, and inferior results compared with non-ARMD revisions [10, 19, 23]. Subsequently, surgeons and worldwide regulatory authorities widely recommended early revision for ARMD with a lower surgical threshold adopted [7, 10, 14, 18, 28]. Given the high revision rates of MoMHR [5, 14, 20] coupled with the fact that many patients undergoing MoMHR will undergo regular patient surveillance over future years [15, 18, 28], it is expected that a large proportion of this young and active patient population is likely to require revision surgery for ARMD. Therefore, it is important for surgeons to have robust information that they can use to counsel patients about the risk of complications and reoperation after this unanticipated revision indication compared with patients undergoing MoMHR revisions for non-ARMD indications, which represent the conventional modes of arthroplasty revision. However, large cohort studies comparing these different revision indications are lacking. We observed patients undergoing MoMHR revision for ARMD had approximately half the risk of rerevision and death compared with matched patients undergoing non-ARMD revisions; however, infected MoMHR revisions were responsible for the increased mortality risk observed after non-ARMD revision. Non-ARMD revisions performed for infection and dislocation/subluxation had the lowest implant survival. These findings are clinically important because they are contradictory to earlier observations, namely that MoMHR revisions performed for ARMD had a higher risk of complications and reoperation compared with non-ARMD revisions [10, 19, 23].

This study has a number of limitations. First, using observational data means, we cannot infer causality. Second, our inclusion criteria may have introduced selection bias. Excluding incompletely recorded staged revisions may have affected reporting of the study endpoints in non-ARMD revisions because most staged revisions are performed for infection. We also excluded non-ARMD revisions with “other” indications, which may have represented undiagnosed or incorrectly recorded ARMD, especially during the early years [10, 13, 21]. Although this provided a more robust cohort for analysis, the earliest and most severe ARMD revisions with the highest risk of complications and reoperation [10] may have been excluded. However, using linked data from the world’s largest arthroplasty registry ensures adequate statistical power and reporting on an unselected population using robust statistical methods, including propensity score matching, minimizes the potential for confounding by revision indication (analysis in unmatched patients demonstrated different results; see Appendix, Supplemental Digital Content 3). Third, the non-ARMD group comprised a heterogeneous collection of revision indications, which could feasibly range from simple problems (for example, early component loosening with no bone loss) to complex ones (such as infection with severe osteolysis). All non-ARMD indications were grouped together for this study because (1) these represent all modes of conventional hip arthroplasty revision, whereas ARMD represents an almost unique mode of revision in MoMHRs [5, 20]; and (2) the initial MoMHR revisions performed for ARMD were associated with an increased risk of early complications and reoperation and inferior patient-reported outcome scores compared with revisions performed for non-ARMD indications, which subsequently led to the widespread recommendation of early revision for ARMD with a lower surgical threshold adopted [10]. Nevertheless, it is recognized that depending on the proportion of different revision indications and procedural complexities in the non-ARMD group, the heterogeneous non-ARMD group could potentially lead to incorrect conclusions being drawn about the different study endpoints in non-ARMD revisions relative to ARMD revisions. For this reason, sensitivity analysis was performed in which MoMHR revisions performed for infection were excluded with the findings from this analysis discussed subsequently.

A fourth limitation was that despite using multiple imputation, missing data for some variables (namely BMI) may have affected the findings. However, sensitivity analysis based on the complete data set (excluding BMI) produced similar findings and effect sizes (see Appendix, Supplemental Digital Content 1). Fifth, despite matching, there is potential for residual confounding. However, the observed effect sizes were large; therefore, it is unlikely any unmeasured factors would be large enough to change the direction of the effect sizes. Matching may also have reduced the generalizability of our findings given that almost half of the unmatched cohort was excluded from the matched analysis. Many pre-2012 revisions were excluded after matching, which may represent patients with the most difficult and aggressive ARMD who experienced the highest risk of complications and reoperation [10, 19, 23]. However, the direction of the effect sizes for all study endpoints was the same in the unmatched and matched cohorts (see Appendix, Supplemental Digital Content 3).

A sixth limitation was that registries do not record histopathologic and microbiologic results from intraoperative samples; therefore, in some cases, the revision diagnoses recorded by surgeons may have changed after sample analysis. Seventh, although further surgery represents an important endpoint to consider after revision, the NJR does not collect data on nonrevision procedures such as those performed for dislocations (closed reductions), infections (débridement and washout), and periprosthetic fractures (internal fixation). Eighth, registries potentially underreport revisions [24], although there is no reason to suspect that underreporting would differ between groups. Ninth, it is recognized that patient followup after MoMHR revision surgery was likely to be less stringent than the regular patient surveillance used before revision [18, 28]. This may have also led to underreporting of rerevisions after both ARMD and non-ARMD revision procedures, which would mean that the implant survival rates observed in the present study represent a best case scenario for both groups. Finally, it is feasible that both patients and surgeons might be increasingly reluctant to undertake rerevision surgery even if the revision arthroplasty was not functioning well. Although this would similarly result in the underreporting of rerevisions, there is no reason to suspect that the magnitude of underreported rerevisions would differ between the revision groups.

There were no differences in the risk of intraoperative complications between matched ARMD and non-ARMD revisions with femoral fractures representing the most common complications as reported during conventional THA revisions [1]. We are unaware of any studies specifically reporting on intraoperative complications after MoMHR revision surgery performed for different revision indications. This may be because these events are considered rare and require large cohort studies, which are lacking. However, it is important to define the risks given the increasing burden of MoMHR revision surgery [7, 20]. The risk of intraoperative periprosthetic femoral fractures in both ARMD and non-ARMD revisions was lower than in previous studies [1], although this may reflect the present cohort including many revision procedures in which the femoral component was retained, and also including revisions of hip resurfacings, which are designed to conserve femoral bone stock. The potentially destructive nature of ARMD lesions can lead to significant bone defects, which can require major reconstruction [10, 13, 17]. The low and similar risk of intraoperative periprosthetic femoral fractures in ARMD and non-ARMD revisions in the present large cohort is therefore somewhat reassuring.

Although patients undergoing ARMD revisions had half the risk of death compared with all non-ARMD revisions, there was no longer a difference in the mortality risk between the groups when revisions performed for infection were excluded. This suggests that infected MoMHR revisions were responsible for the increased risk of mortality initially observed after non-ARMD revision. In non-MoMHR revisions, the risk of mortality is higher when procedures are performed for periprosthetic infection compared with aseptic indications [6, 30]. This difference can persist for up to 5 years after revision with the risk of mortality after revision for periprosthetic infection being similar to some common cancers [30]. However, the reasons for the increased mortality associated with infected revisions are complex and multifactorial [22]. The present findings regarding an increased mortality risk after MoMHR revisions for infection therefore are similar to observations in non-MoMHR revisions performed for infection. However, we are unaware of any other studies reporting mortality rates after MoMHR revision surgery performed for different revision indications.

The observation that ARMD revisions have half the risk of rerevision compared with non-ARMD revisions is contradictory to previous observations with this finding persisting even when the revisions for infection were removed from the non-ARMD group. Numerous small studies have reported an increased risk of early complications and reoperation after their initial experience with ARMD revision surgery [10, 16, 19, 23] with an increased early risk of rerevision compared with non-ARMD indications [10]. However, these studies had numerous limitations that may explain the differences between their findings and ours; those earlier studies were smaller, often underpowered, single-center cohorts perhaps influenced by surgeon inexperience (first MoMHR revisions performed) with the potential for confounding by revision indication. The Australian Joint Registry recently reported high rerevision rates after 884 MoMHR revisions with rerevision not influenced by type of revision performed or bearing surface implanted [8, 29]. However, important limitations included reporting on a relatively small cohort (with < 200 ARMD revisions), excluding stemmed MoM THAs, including the initial MoMHR revisions performed, which may influence the findings, not stratifying the cohort by revision indication, and only reporting rerevision rates [8, 29]. Comparing our 5-year implant survival rates (ARMD = 94.3%; non-ARMD = 90.5%) with those after revision of conventional THAs in the NJR (88%-89% depending on bearing and fixation) [20] suggests that matched non-ARMD revisions have similar or better implant survival as conventional THA revisions. The implant survival rates after ARMD revision in this study were certainly much improved compared with those reported for the earliest ARMD revisions (5-year implant survival rate of 56%) [16]. We suspect that the reduced rerevision risk observed after ARMD revision in our study relates to increased awareness of this problem, regular patient surveillance, and the widespread adoption of early surgery and a lower threshold for performing ARMD revisions following recommendations from worldwide regulatory authorities and surgeons [7, 10, 14, 15, 18, 28]. Even when acknowledging our limitations, we can conclude that ARMD revision surgery does not appear to cause any additional morbidity compared with non-ARMD revisions. The lower rerevision rates after ARMD revision therefore provide some reassurance to both surgeons and patients given the extremely poor prognosis initially reported [7, 10, 19, 23]. However, surveillance bias of patients after MoMHR (more regular patient followup, additional investigation with blood metal ions and cross-sectional imaging, and performing revision surgery for ARMD at an earlier stage than previously) is also likely to have contributed to the increasing rate of ARMD revision surgery with extended followup [14]. Therefore, some MoMHR revisions may actually be performed too early, which has the potential for surgical risk outweighing any benefits. Although research is needed to refine the thresholds for performing ARMD revision surgery, we consider the threshold for performing ARMD revision need not be lowered further.

Although many non-ARMD indications (loosening and/or lysis, fracture, unexplained pain) had inferior implant survival compared with ARMD revisions, MoMHRs revised for infection and dislocation/subluxation had the poorest implant survival. Managing periprosthetic joint infection is challenging with a high risk of complications and reoperation reported after conventional THA revisions for infection [6, 22]. This study confirms the same is true for infected MoMHR revisions. Contrary to other bearing surfaces in which infected revisions occur early, the revision risk for infection continues to increase with time since implantation in MoMHR [20]. Although the reasons for this remain unknown, this is concerning given that many MoMHRs may undergo future revision for infection and the poor prognosis reported here for this subgroup. Dislocation is uncommon after large-diameter MoMHR [14, 19], but if it occurs, our findings suggest rerevision rates are high after revision. We suspect that controlling hip instability in MoMHRs is challenging and likely to be multifactorial with some hips requiring a reduction in femoral head size at revision. The higher rerevision rates observed in non-ARMD revisions are concerning given many such revisions continue to be performed [5, 20]. Our work provides surgeons with evidence that can be used to counsel and inform patients about the substantial risks associated with non-ARMD revision surgery, especially when performed for infection or dislocation/subluxation.

It is worth acknowledging that 19% of rerevisions performed in the whole matched cohort were performed for ARMD, which appears high. Recurrence of ARMD has been frequently reported after MoMHR revision surgery performed for both ARMD and non-ARMD indications [16, 17]. The present study observed that rerevisions for ARMD were not the result of initially revising to another MoM bearing surface given that this happened in only a few patients (Table 1) of which none subsequently required rerevision for ARMD. However, because we analyzed registry data, it is only possible to speculate reasons for ARMD rerevision, which include incomplete ARMD lesion débridement (either intentional or unintentional), revision to a construct that includes a MoM junction (such as a cobalt-chrome femoral head on a titanium alloy femoral taper despite a metal-on-polyethylene bearing surface), or possible misdiagnosis of the initial revision indication [17].

Contrary to previous observations, this large nationwide study observed that patients undergoing MoMHR revision surgery for ARMD have approximately half the risk of rerevision compared with matched non-ARMD revisions. We suspect worldwide regulatory authorities have positively influenced rerevision rates after ARMD revision by promoting regular patient surveillance and recommending that surgeons exercise a lower revision threshold. We conclude that performing early revision surgery for ARMD has not been associated with additional morbidity compared with non-ARMD revisions and consider the threshold for performing ARMD revision surgery need not be lowered further. The high risk of rerevision after MoMHR revisions performed for infection and dislocation is concerning and requires further investigation. Infected MoMHR revisions were responsible for the increased mortality risk observed after non-ARMD revision, which parallels findings in infected non-MoMHR revisions.

## References

[1] Abdel MP Houdek MT Watts CD Lewallen DG Berry DJ Epidemiology of periprosthetic femoral fractures in 5417 revision total hip arthroplasties: a 40-year experience. Bone Joint J. 2016;98:468–474. 27037428 10.1302/0301-620X.98B4.37203

[2] Austin PC Balance diagnostics for comparing the distribution of baseline covariates between treatment groups in propensity-score matched samples. Stat Med. 2009;28:3083–3107. 19757444 10.1002/sim.3697 PMC3472075

[3] Austin PC Some methods of propensity-score matching had superior performance to others: results of an empirical investigation and Monte Carlo simulations. Biom J. 2009;51:171–184. 19197955 10.1002/bimj.200810488

[4] Austin PC The performance of different propensity score methods for estimating marginal hazard ratios. Stat Med. 2013;32:2837–2849. 23239115 10.1002/sim.5705 PMC3747460

[5] Australian Orthopaedic Association National Joint Replacement Registry: hip and knee arthroplasty. Annual report 2015. Available at: https://aoanjrr.sahmri.com/annual-reports-2015. Accessed November 23, 2016.

[6] Berend KR Lombardi AV Jr Morris MJ Bergeson AG Adams JB Sneller MA Two-stage treatment of hip periprosthetic joint infection is associated with a high rate of infection control but high mortality. Clin Orthop Relat Res. 2013;471:510–518. 22983683 10.1007/s11999-012-2595-x PMC3549176

[7] De Smet KA Van Der Straeten C Van Orsouw M Doubi R Backers K Grammatopoulos G Revisions of metal-on-metal hip resurfacing: lessons learned and improved outcome. Orthop Clin North Am. 2011;42:259–269. 21435500 10.1016/j.ocl.2011.01.003

[8] de Steiger RN Miller LN Prosser GH Graves SE Davidson DC Stanford TE Poor outcome of revised resurfacing hip arthroplasty. Acta Orthop. 2010;81:72–76. 20170416 10.3109/17453671003667176 PMC2856207

[9] Glynn RJ Schneeweiss S Stürmer T Indications for propensity scores and review of their use in pharmacoepidemiology. Basic Clin Pharmacol Toxicol. 2006;98:253–259. 16611199 10.1111/j.1742-7843.2006.pto_293.x PMC1790968

[10] Grammatopoulos G Pandit H Kwon YM Gundle R McLardy-Smith P Beard DJ Murray DW Gill HS Hip resurfacings revised for inflammatory pseudotumour have a poor outcome. J Bone Joint Surg Br. 2009;91:1019–1024. 19651827 10.1302/0301-620X.91B8.22562

[11] Jameson SS Baker PN Mason J Porter ML Deehan DJ Reed MR Independent predictors of revision following metal-on-metal hip resurfacing: a retrospective cohort study using National Joint Registry data. J Bone Joint Surg Br. 2012;94:746–754. 22628587 10.1302/0301-620X.94B6.29239

[12] Jameson SS Baker PN Mason J Rymaszewska M Gregg PJ Deehan DJ Reed MR Independent predictors of failure up to 7.5 years after 35,386 single-brand cementless total hip replacements: a retrospective cohort study using National Joint Registry data. Bone Joint J. 2013;95:747–757. 23723267 10.1302/0301-620X.95B6.31378

[13] Langton DJ Jameson SS Joyce TJ Hallab NJ Natu S Nargol AV Early failure of metal-on-metal bearings in hip resurfacing and larger-diameter total hip replacement: a consequence of excess wear. J Bone Joint Surg Br. 2010;92:38–46. 20044676 10.1302/0301-620X.92B1.22770

[14] Matharu GS Judge A Murray DW Pandit HG Prevalence of and risk factors for hip resurfacing revision: a cohort study into the second decade after the operation. J Bone Joint Surg Am. 2016;98:1444–1542. 27605688 10.2106/JBJS.15.01234

[15] Matharu GS Mellon SJ Murray DW Pandit HG Follow-up of metal-on-metal hip arthroplasty patients is currently not evidence based or cost effective. J Arthroplasty. 2015;30:1317–1323. 25861918 10.1016/j.arth.2015.03.009

[16] Matharu GS Pandit HG Murray DW Poor survivorship and frequent complications at a median of 10 years after metal-on-metal hip resurfacing revision. Clin Orthop Relat Res. 2017;475:304–314. 27188835 10.1007/s11999-016-4882-4 PMC5213920

[17] Matharu GS Pynsent PB Dunlop DJ Revision of metal-on-metal hip replacements and resurfacings for adverse reaction to metal debris: a systematic review of outcomes. Hip Int. 2014;24:311–320. 24970319 10.5301/hipint.5000140

[18] Medical and Healthcare products Regulatory Agency (MHRA). Medical Device Alert: all metal-on-metal (MoM) hip replacements. 2012 MDA/2012/036. Available at: https://www.gov.uk/drug-device-alerts/medical-device-alert-metal-on-metal-mom-hip-replacements-updated-advice-with-patient-follow-ups. Accessed November 23, 2016.

[19] Munro JT Masri BA Duncan CP Garbuz DS High complication rate after revision of large-head metal-on-metal total hip arthroplasty. Clin Orthop Relat Res. 2013;472:523–528. 10.1007/s11999-013-2979-6 PMC3890190 23572349

[20] National Joint Registry for England, Wales, Northern Ireland and the Isle of Man. 13th annual report 2016. Available at: http://www.njrcentre.org.uk/njrcentre/Portals/0/Documents/England/Reports/13th%20Annual%20Report/07950%20NJR%20Annual%20Report%202016%20ONLINE%20REPORT.pdf. Accessed November 23, 2016.

[21] Pandit H Glyn-Jones S McLardy-Smith P Gundle R Whitwell D Gibbons CL Ostlere S Athanasou N Gill HS Murray DW Pseudotumors associated with metal-on-metal hip resurfacings. J Bone Joint Surg Br. 2008;90:847–851. 18591590 10.1302/0301-620X.90B7.20213

[22] Parvizi J Haddad FS Periprosthetic joint infection: the last frontier. Bone Joint J. 2015;97:1157–1158. 26330578 10.1302/0301-620X.97B9.37018

[23] Rajpura A Porter ML Gambhir AK Freemont AJ Board TN Clinical experience of revision of metal on metal hip arthroplasty for aseptic lymphocyte dominated vasculitis associated lesions (ALVAL). Hip Int. 2011;21:43–51. 21279962 10.5301/hip.2011.6276

[24] Sabah SA Henckel J Cook E Whittaker R Hothi H Pappas Y Blunn G Skinner JA Hart AJ Validation of primary metal-on-metal hip arthroplasties on the National Joint Registry for England, Wales and Northern Ireland using data from the London Implant Retrieval Centre: a study using the NJR dataset. Bone Joint J. 2015;97:10–18. 25568407 10.1302/0301-620X.97B1.35279 PMC4548488

[25] Sabah SA Henckel J Koutsouris S Rajani R Hothi H Skinner JA Hart AJ Are all metal-on-metal hip revision operations contributing to the National Joint Registry implant survival curves? A study comparing the London Implant Retrieval Centre and National Joint Registry datasets. Bone Joint J. 2016;98:33–39. 26733513 10.1302/0301-620X.98B1.36431 PMC4714035

[26] Smith AJ Dieppe P Howard PW Blom AW; National Joint Registry for England and Wales. Failure rates of metal-on-metal hip resurfacings: analysis of data from the National Joint Registry for England and Wales. Lancet. 2012;380:1759–1766. 23036895 10.1016/S0140-6736(12)60989-1

[27] Smith AJ Dieppe P Vernon K Porter M Blom AW; National Joint Registry of England and Wales. Failure rates of stemmed metal-on-metal hip replacements: analysis of data from the National Joint Registry for England and Wales. Lancet. 2012;379:1199–1204. 22417410 10.1016/S0140-6736(12)60353-5

[28] US Food and Drug Administration. Medical devices. Metal-on-metal hip implants. Information for orthopaedic surgeons. 2013. Available at: http://www.fda.gov/MedicalDevices/ProductsandMedicalProcedures/ImplantsandProsthetics/MetalonMetalHipImplants/ucm241667.htm. Accessed November 23, 2016.

[29] Wong JM Liu YL Graves S de Steiger R What is the rerevision rate after revising a hip resurfacing arthroplasty? Analysis from the AOANJRR. Clin Orthop Relat Res. 2015;473:3458–3464. 25721576 10.1007/s11999-015-4215-z PMC4586194

[30] Zmistowski B Karam JA Durinka JB Casper DS Parvizi J Periprosthetic joint infection increases the risk of one-year mortality. J Bone Joint Surg Am. 2013;95:2177–2184. 24352771 10.2106/JBJS.L.00789

